# Propofol attenuates sepsis-induced acute kidney injury by regulating miR-290-5p/CCL-2 signaling pathway

**DOI:** 10.1590/1414-431X20187655

**Published:** 2018-10-11

**Authors:** Guodong Zheng, Hong Qu, Fen Li, Weiquan Ma, Hong Yang

**Affiliations:** 1 Department of Critical Care Medicine, Third Affiliated Hospital of Southern Medical University, Guangzhou, China; 2 Department of Hematology, Guangzhou Panyu Central Hospital, Guangzhou, China

**Keywords:** Propofol, Sepsis, miR-290-5p, CCL-2, Acute kidney injury

## Abstract

Previous studies have indicated that propofol has immunomodulatory and antioxidative properties. However, the renoprotection effect and the precise mechanisms of propofol in sepsis-induced renal injury remain unclear. The purpose of the present study was to investigate the role of miR-290-5p/CCL-2 signaling in septic mice treatment with propofol. Mice were treated with propofol (50 mg/kg) twice within 24 h. Survival outcome was monitored within 48 h. The mRNA and protein levels were assayed by qRT-PCR and western blotting, respectively. Mouse podocytes (MPC5) were treated with lipopolysaccharide (LPS) to establish the cell model *in vitro*. The proliferation of MPC5 was monitored using the MTS assay. Cell apoptosis was analyzed by flow cytometry. Propofol improved survival outcome and alleviated acute kidney injury in cecal ligation and puncture-operated mice. Propofol increased miR-290-5p expression and decreased CCL-2 and inflammatory cytokines levels in the kidney for septic mice. We found that miR-290-5p was a direct regulator of CCL-2 in MPC5. Propofol could abrogate LPS-induced growth inhibition and apoptosis in MPC5. Meanwhile, propofol inhibited CCL-2 expression in LPS-treated MPC5, however, knockdown of miR-290-5p abrogated the inhibitory effect propofol on the mRNA and protein expressions of CCL-2. Propofol could serve as an effective therapeutic medication to suppress sepsis-induced renal injury *in vivo* and *in vitro* by regulating the miR-290-5p/CCL-2 signaling pathway.

## Introduction

Sepsis is a variety of serious infectious diseases with pathogens that can release bacterial toxin into the body and induce an inflammatory response in the host, leading to septic shock and multiple organ failure (1). Acute kidney injury (AKI) is associated with approximately 70% mortality triggered by sepsis in the intensive care unit (ICU) (2). In addition, sepsis-associated AKI is associated strongly with a poor prognosis and poses significant clinical challenges for clinicians (3). To our knowledge, there is no effective therapeutic medication to improve the clinical outcomes of sepsis-associated AKI (4). Therefore, we need to explore novel drugs to protect the kidney from sepsis. Propofol has long been recognized as a rapid, short-acting intravenous anesthetic, widely used in clinical anesthesia as well as for sedation in the ICU (5). Recently, propofol has been reported to improve oxidative stress and inflammatory response in various tissues and organs, including lung, brain, and liver (6–9). In addition, propofol shows renoprotective effects in endotoxemia (10), ischemia-reperfusion (11), and orthotopic liver transplantation-induced AKI (12). In septic animal models, propofol treatment can protect the kidney from sepsis-induced AKI by decreasing inflammatory cytokines and inhibiting oxidative stress (13). However, potential molecular mechanisms of propofol in cecal ligation and puncture (CLP)-induced AKI have not been clearly delineated. A class of small non-coding RNAs (18–25 nucleotides), known as microRNAs (miR), have emerged as the post-translational modulators that regulate the translation of target messenger RNAs (mRNAs) by binding to its 3′-untranslated regions (3′-UTRs) (14). miRs are involved in a wide variety of diseases, including sepsis-associated AKI (15). Gene ontology (GO) analysis indicates that differentially expressed miRs in sepsis-induced AKI are primarily related to regulation of oxidative stress and mitochondrial dysfunction pathways (16). miRs also modulate the inflammatory response to endotoxin in mice (17). For example, miR27a is up-regulated and promotes inflammatory response in sepsis (18). miR-205-5b shows an anti-inflammatory activity in lipopolysaccharide (LPS)-induced sepsis (19). In the present study, we aimed to determine the anti-inflammatory activity of propofol associated with miR-mediated post-transcriptional regulatory mechanism.

## Material and Methods

### Animal treatment

A total of 48 male 8-week-old C57BL/6J mice (body weight 20±2 g) were obtained from the Third Affiliated Hospital of Southern Medical University (China) and allowed to acclimate to the environment for 1 week. The mice were given free access to food and tap water and were individually caged under controlled temperature (23±2°C) and humidity (55±5%) with an artificial 12-h light/dark cycle. Sepsis in mice was induced by CLP surgical operation, as described previously (13). To investigate the effect of propofol during sepsis-induced AKI, the mice were randomly divided into 4 groups as follows: i) sham-operated mice (n=12) as control group, ii) CLP group (n=12) injected with normal saline, iii) propofol group (n=12) that received propofol injection (50 mg/kg; twice within 24 h; Sigma-Aldrich, USA) in sham-operated mice, and iv) propofol+CLP group that received propofol injection (50 mg/kg; twice within 24 h) in CLP-operated mice (n=12). Twenty-four hours after CLP surgical operation, mice were sacrificed by an overdose of sodium pentobarbital (2%; 200 mg/kg; Sigma-Aldrich). The blood from the hearts was collected for serum biochemical analysis. Kidneys were collected and immediately frozen in liquid nitrogen and 4% formalin at room temperature for gene and protein analysis and paraffin-embedded histological analysis, respectively. In another experiment, we observed the 48-h survival of CLP mice with or without propofol treatment (n=12 in each group). This experiment was approved by the Ethics Committee of the Third Affiliated Hospital of Southern Medical University.

### Enzyme-linked immunosorbent assay (ELISA)

Serum CCL-2 was measured by a mouse ELISA kit (Cat. No. E-EL-M0006c; Elabscience Biotechnology Co., Ltd, China). Glutamic oxaloacetic transaminase (GOT) (Cat. No. c010-1) and glutamic pyruvic transaminase (GPT) (Cat. No. c009-1) were determined using assay kits (Nanjing Jiancheng Biology Engineering Institute, China). In brief, 5 μL serum and 25 μL GOT or GPT substrates were added to 96-well plates at 37°C for 30 min. Then, 2,4-dinitrophenylhydrazine (25 μL) was added to all wells at 37°C for 30 min. Finally, sodium hydroxide (0.4 mol/L; 250 μL) was added to stop the reactions at room temperature for 15 min, and the absorbance was measured at 510 nm. Serum creatinine (Cre; Cat. No. C011-1; Nanjing Jiancheng Biology Engineering Institute, China) and blood urea nitrogen (BUN; Cat. No. C013-2; Nanjing Jiancheng Biology Engineering Institute) levels were measured using an autoanalyzer and an enzymatic kinetic method using commercial kits following the manufacturer’s protocol with a SpectraMax M5 ELISA plate reader (Molecular Devices, LLC, USA).

### Hematoxylin & eosin (H&E) staining

Kidneys were collected and fixed with 4% formalin at room temperature for 24 h, and paraffin-embedded. Tissues were cut into 3-μm thick sections, which were stained with H&E (Beyotime Institute of Biotechnology, China) at room temperature and visualized under a microscope (Leica DM 2500; Leica Microsystems GmbH, Germany). Renal injury was assessed using a previously described 0–4 scale (20), in which 0 is none; 1, <10%; 2, 10–25%; 3, 26–75%; or 4, >75%.

### RNA analysis and RT-qPCR

Total RNA was extracted by TRIzol (Invitrogen, USA) according to the manufacturer’s protocol. cDNA was synthesized by reverse transcription reactions with 2 μg of total RNA using Moloney murine leukemia virus reverse transcriptase (Invitrogen; Thermo Fisher Scientific, USA) according to the manufacturer’s protocol. PCR reaction mixtures (20 µL) were prepared using the TaqMan Universal PCR Master Mix (Thermo Fisher Scientific) and performed using a DNA Engine (ABI 7300; Thermo Fisher Scientific). The reaction conditions were set according to the manufacturer’s protocol. The Cq (quantification cycle fluorescence value) was calculated using SDS software, version 2.1 (Applied Biosystems; Thermo Fisher Scientific), and the relative expression levels of miR and mRNA were calculated using the 2^-ΔΔCt^ method (21) and normalized to the internal control U6 and glyceraldehyde 3-phosphate dehydrogenase (GAPDH), respectively. The primers were synthesized by Sangon Biotech (China) as shown in Table 1. The PCR products were confirmed by 2% agarose (Sigma-Aldrich) gel electrophoresis and visualized under a gel imaging analysis system (Bio-Rad Laboratories, Inc., USA).

**Table 1.** Primers for RT-qPCR.

Gene	Forward primer (5′-3′)	Reverse primer (5′-3′)
miR-124	TCGGCAGGTAAGGCACGCGGTG	TCAACTGGTGTCGTGGAGTCGGC
miR-290-5p	GCTGGGTTTCACGGGGGTATCAA	TCAACTGAGTGCCGTAGGGTGCG
miR-292-5p	AGCTGGGGTTTTCUCGGGGGUC	GACGTTGAGTCCGATGTACCCGTA
U6	CTCGCTTCGGCAGCACATATACT	ACGCTTCACGAATTTGCGTGTC
CCL-2	ATTTCCACACTTCTATGCCTCCT	ATCCAGTATGGTCCTGAAGATCA
IL-1β	ACCTTCCAGGATGAGGACATGA	CTAATGGGAACGTCACACACCA
TNF-α	TACTCCCAGGTTCTCTTCAAGG	GGAGGTTGACTTTCTCCTGGTA
NGAL	GATGTTGTTATCCTTGAGGCCC	CACTGACTACGACCAGTTTGCC
GAPDH	GCACCGTCAAGCTGAGAAC	TGGTGAAGACGCCAGTGGA

### Cell culture

Mouse podocytes (MPC5) were obtained from the National Infrastructure of Cell Line Resource (Serial number: 3111C0001CCC000230; China) and maintained in RPMI-1640 (Invitrogen, USA) supplemented with 10% FBS (Invitrogen) at 37°C in a humidified incubator (Thermo Fisher Scientific), under 5% CO_2_, 95% air atmosphere. MPC5 were treated with lipopolysaccharide (LPS; 100 ng/mL, Sigma-Aldrich), or combined with propofol (100 μM) or miR-290-5p inhibitors (100 nM). All of the experiments were performed in triplicate.

### Transfection with miR-290-5p mimics and inhibitors

The sequences of the miR-290-5p mimics (5′-ACUCAAACUAUGGGGGCACUUU-3′) and anti-miR-290-5p (antisense inhibitor of miR-290-5p: 5′-AAAGUGCCCCCAUAGUUUGAGU-3′) were synthesized by RiboBio (China). The MPC5 were transfected using Lipofectamine 2000 (Invitrogen; Thermo Fisher Scientific) at a final concentration of 100 nM. At 48 h post-transfection, the cells were harvested for analysis.

### Luciferase reporter gene assay

The potential binding sites between miR-290-5p and CCL-2 were obtained using online prediction software (miRanda-mirSVR; http://www.microrna.org/), miRDB (http://www.mirdb.org/), and TargetScan (http://www.targetscan.org/). The wild-type (WT) and mutant-type (MUT) 3′-UTR of CCL-2 were synthesized by RiboBio (China) and inserted into the multiple cloning sites of the luciferase expressing pMIR-REPORT vector (Ambion; Thermo Fisher Scientific, Inc.). For the luciferase assay, MPC5 (1×10^5^) was seeded into 24-well plates and co-transfected with luciferase reporter vectors containing the WT and MUT of CCL-2-3′-UTR (0.5 μg) and mimics and inhibitors of miR-290-5p (100 nM) using Lipofectamine 2000 (Invitrogen; Thermo Fisher Scientific). Luciferase activity was measured using the Dual Luciferase Reporter® Assay System (Cat. No. E1960; Promega, USA) on a Luminoskan^TM^ Ascent Microplate Luminometer (Thermo Fisher Scientific).

### MTS assay

The proliferation of MPC5 was monitored using the MTS assay kit (Promega Corporation, USA). Absorbance was measured at 492 nm using an ELISA reader (MD SpectraMax M5; Molecular Devices, LLC, USA).

### Flow cytometry analysis

MPC5 was treated with LPS (100 ng/mL), LPS (100 ng/mL) + propofol (100 μM), or LPS (100 ng/mL) + propofol (100 μM) + miR-290-5p inhibitors (100 nM) for 24 h. Cells were collected after digestion and were washed twice with PBS and centrifuged at 1200 *g* for 5 min at 4°C. The supernatant was discarded, and the cells were then resuspended and fixed in ice-cold 75% ethanol and stored at 4°C. Annexin V-FITC apoptosis detection kit was purchased from Invitrogen. The samples were analyzed using flow cytometer (BD Biosciences, USA). The data were processed by Cell Quest Software (version 5.1, BD Biosciences,).

### Western blotting

Proteins were extracted with radio immunoprecipitation assay (RIPA) buffer (Cat. No. P0013B; Beyotime Institute of Biotechnology), and the concentrations were determined using the Bicinchoninic Acid Kit for Protein Determination (Cat. No. BCA1-1KT; Sigma-Aldrich; Merck KGaA). Protein (30 μg) for each sample was separated on a 10% SDS-PAGE gel and transferred to nitrocellulose membranes (Bio-Rad Laboratories, Inc., USA). The membranes were incubated with the primary antibody CCL-2 (Cat. No. sc-1784; dilution: 1: 1,000; Santa Cruz Biotechnology, USA), and NF-κB/p65 (Cat. No. 3034; dilution: 1: 500; Cell Signaling Technology, Inc., USA). Following three washes with TBST, the membranes were incubated with the appropriate horseradish peroxidase-conjugated secondary antibody (Cat. No. sc-516102; dilution: 1:10,000; Santa Cruz Biotechnology) at room temperature for 2 h and visualized by chemiluminescence (Thermo Fisher Scientific, Inc.). Signals were analyzed with Quantity One® software version 4.5 (Bio Rad Laboratories, Inc.). GAPDH (Cat. no: 2118; dilution: 1: 2,000; Cell Signaling Technology, Inc., USA) and histone (Cat. no: 9715; dilution: 1: 2,000; Cell Signaling Technology, Inc.) were used as control antibodies.

### Statistical analysis

Data are reported as the mean±SD for each group. All statistical analyses were performed using PRISM version 5.0 (GraphPad Software, Inc., USA). Statistical differences between two groups were determined using Student’s *t*-test. Inter-group differences were analyzed by one-way analysis of variance, followed by a *post-hoc* Tukey’s test for multiple comparisons. Survival rates were calculated using the Kaplan-Meier method with the log-rank test applied for comparison. P<0.05 was considered to indicate a statistically significant difference.

## Results

### Propofol had a significant effect on sepsis-induced AKI in mice

**Figure 1.**
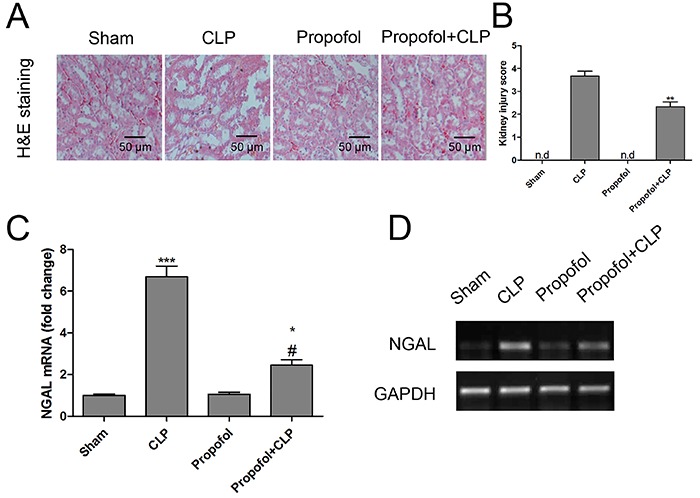
Propofol attenuated cecal ligation and puncture (CLP)-induced acute kidney injury in mice. Twenty-four hours after CLP surgical operation, renal histology (magnification 200×; *A*) and injury score (*B*) were performed in the kidneys from mice with or without propofol treatment. Renal expression of neutrophil gelatinase associated lipocalin (NGAL) mRNA (relative to the controls) was measured by RT-qPCR (*C*) and verified by agarose gel electrophoresis analysis (*D*). Data are reported as means±SD (n=12 per group). *P<0.05, **P<0.01, ***P<0.001 compared with the control group; ^#^P<0.05 compared with the CLP group (ANOVA). nd: not detected.

In our study, CLP surgical operation was performed to establish the polymicrobial sepsis-induced AKI in mice. Twenty-four hours after CLP surgical operation, the serum levels of GOT, GPT, BUN, and Cre were significantly higher in CLP-operated mice than in the control group, while propofol treatment decreased those levels in CLP-operated mice (Table 2). In addition, H&E staining was performed to observe the extent of renal injury. As indicated in Figure 1A and B, the severe architectural disruptions of kidney were triggered by CLP surgical operation, including tubular dilatation and brush border loss. Renal injury scores were significantly increased in the CLP group compared with the control group. In contrast, propofol treatment preserved the morphologic integrity of kidney in CLP-operated mice. Neutrophil gelatinase associated lipocalin (NGAL) is a highly predictive biomarker of AKI (22). In the present study, the mRNA expression of NGAL was measured in the kidney from septic mice with or without propofol treatment. The results demonstrated that NGAL mRNA increased by 7-fold after CLP surgical operation, but propofol treatment could reverse the mRNA expression of NGAL induced by CLP in the kidney from mice (Figure 1C). Furthermore, the PCR products of NGAL were confirmed by 2% agarose gel electrophoresis (Figure 1D).

**Table 2.** Effects of propofol on hepatic and renal function in cecal ligation and puncture (CLP)-operated or sham-operated mice.

	Sham	CLP	Propofol	Propofol+CLP
CCL-2 (pg/mL)	136.49±15.27	321.67±48.34*	151.82±16.37	198.61±31.47^#^
GOT (U/L)	67.49±7.15	432.67±78.93*	73.83±10.52	173.56±25.73^#^
GPT (U/L)	38.47±5.35	205.71±36.72*	45.84±6.21	75.49±13.51^#^
BUN (mmol/L)	5.78±0.62	17.27±2.31*	4.89±0.71	9.12±1.68^#^
Cre (μmol/L)	4.31±0.51	54.65±10.95*	5.36±0.47	20.61±4.22^#^

Data are reported as the means±SD. *P<0.05 compared with the Sham group; ^#^P<0.05 compared with the CLP group (ANOVA). GOT: glutamic oxaloacetic transaminase; GPT: glutamic pyruvic transaminase; BUN: blood urea nitrogen; Cre: creatinine.

### Propofol improved survival outcome in CLP-operated mice

**Figure 2.**
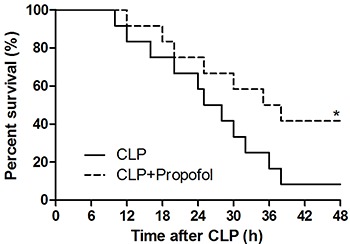
Propofol improved survival outcome in septic mice. Kaplan-Meier survival curves of cecal ligation and puncture (CLP)-operated mice treated or non-treated with propofol. Data are reported as means±SD (n=12 per group). *P<0.05 compared with the CLP group (log-rank test).

We observed the 48-h survival of CLP mice with and without propofol treatment. We found that 92% of mortality occurred within 48 h after CLP surgical operation. However, the septic mice treated with propofol did not die at 38 h, and 42% survived with propofol treatment (Figure 2).

### Propofol inhibited inflammatory gene expression in CLP-operated mice

**Figure 3.**
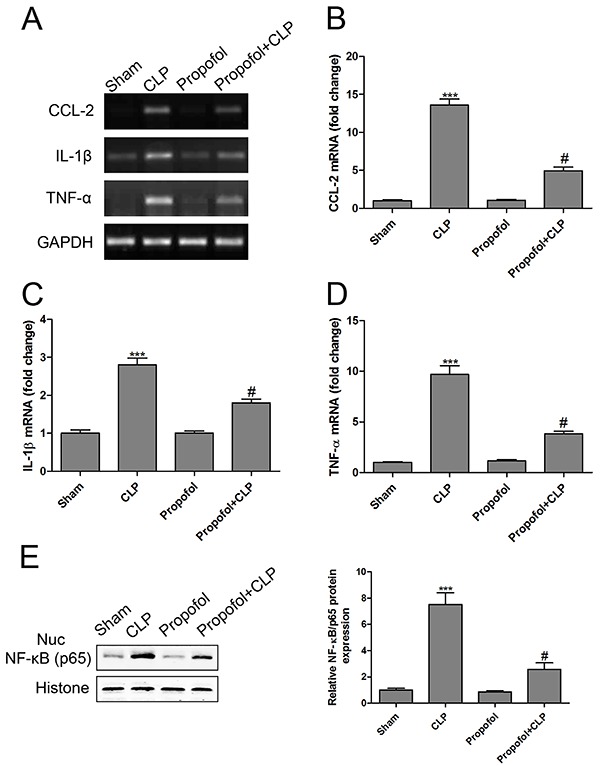
Propofol inhibited excessive inflammatory responses in septic mice. Renal mRNA levels of CCL-2 (*A* and *B*), interleukin (IL)-1β (*A* and *C*), and tumor necrosis factor α (TNF-α) (*A* and *D*) were measured by RT-qPCR and verified by agarose gel electrophoresis analysis. Protein expression of NF-κB (p65) in the nucleus (Nuc) was measured by western blotting (*E*). Data are reported as means±SD (n=12 per group). ***P<0.001 compared with the control group; ^#^P<0.05 compared with the cecal ligation and puncture (CLP) group (ANOVA)

Induction of CCL-2 regulates some inflammatory cytokines levels, such as interleukin 1β (IL-1β), IL-6, and tumor necrosis factor α (TNF-α) (23). In septic mice, CCL-2 induces systemic inflammatory response and promotes tissue repair (24). The plasma concentration of CCL-2 in sepsis patients is significantly higher than in healthy controls (25). In our study, we found that the serum concentration and mRNA of CCL-2 in CLP-operated mice were significantly up-regulated compared to sham-operated mice, while propofol treatment decreased the levels of CCL-2 in septic mice (Table 2, Figure 3A and B). Moreover, septic mice exhibited significantly higher mRNA expression of IL-1β and TNF-α compared with the sham-operated mice. Propofol treatment also inhibited sepsis-induced inflammatory response (Figure 3A, C and D). In addition, we detected the protein expression of NF-Κb (p65) in the nucleus and found an increased NF-κB(p65) level in the kidney from CLP-operated mice, while propofol had the capacity to reduce CLP-induced up-regulation of NF-Κb (p65) protein in the nucleus (Figure 3E). NF-κB as a key transcription factor has been implicated in the process of sepsis-induced inflammatory response. Over-activation of NF-κB is associated with cytoplasmic degradation of its inhibitor IκBα, which leads to the translocation of p65, a subunit of NF-κB, into the nucleus that binds to DNA and enhances the expression of inflammatory cytokines (26).

### Propofol was associated with post-transcriptional regulatory mechanism in septic mice

**Figure 4.**
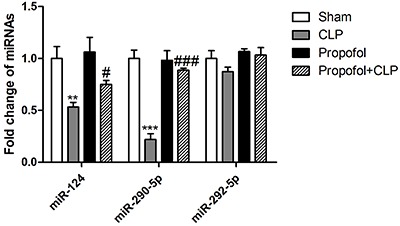
Effects of propofol 24 h after cecal ligation and puncture (CLP) on renal expression of miR-124, miR-290-5p, and miR-292-5p was measured by RT-qPCR in kidneys. Data are reported as means±SD (n=12 per group). **P<0.01, ***P<0.001 compared with the sham group; ^#^P<0.05, ^###^P<0.001 compared with the CLP group (ANOVA).

Based on the above findings, we concluded that CCL-2 played a significant role in the pathogenesis of sepsis. To investigate whether CCL-2 could be regulated by miRs in sepsis-induced AKI, the online prediction softwares miRanda-mirSVR, miRDB, and TargetScan were used for prediction. We found that miR-124, miR-290-5p, and miR-292-5p were overlapped in at least two databases. Therefore, we measured their expression in the kidney from septic mice with or without propofol treatment. The results demonstrated that NGAL miR-124 and miR-290-5p decreased by 47% and 78%, respectively, after CLP surgical operation, which were restored by propofol treatment in septic mice. However, miR-292-5p had no obvious difference among the four groups (Figure 4). Therefore, we focused on miR-290-5p in our study.

**Figure 5.**
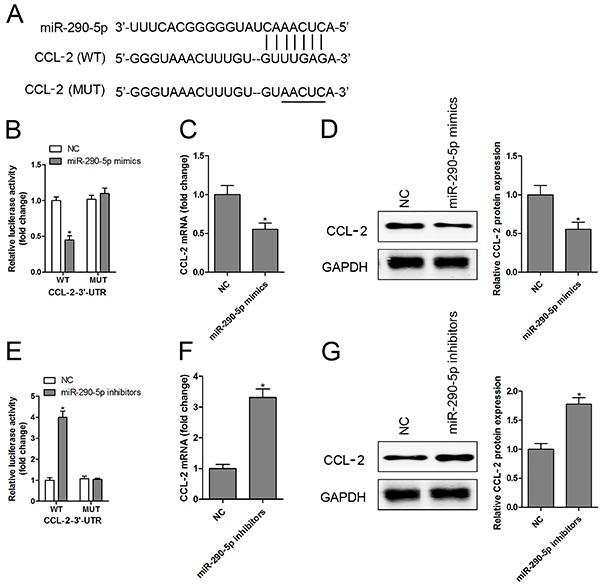
CCL-2 is a direct target gene of miR-290-5p. Schematic representation of the putative miR-290-5p binding site in the 3′-untranslated regions (3′UTR) of CCL-2 was predicted by the online database (*A*). Mouse podocytes cells (MPC5) were co-transfected with the wild type (WT) and mutant (MUT) of CCL-2-3′-UTR and miR-290-5p mimics, and luciferase activity (*B*), mRNA (*C*), and protein (*D*) of CCL-2 were measured. After transfection with the WT and MUT of CCL-2-3′-UTR and miR-290-5p inhibitors, luciferase activity (*E*), mRNA (*F*), and protein (*G*) of CCL-2 were measured. Data are reported as means±SD (n=3 per group). *P*<*0.05 compared with the normal control (NC) group (ANOVA).

First, the conserved binding sites between miR-290-5p and CCL-2 were depicted based on miRanda-mirSVR, miRDB, and TargetScan databases and are shown in Figure 5A. Then, the luciferase reporter plasmids containing WT or MUT 3′-UTR of CCL-2 were constructed. In addition, MPC5 were cotransfected with miR-290-5p mimics and luciferase reporter plasmid. Luciferase reporter assay showed that the luciferase activity of WT 3′-UTR of CCL-2 reduced by nearly 56% with the co-transfection of miR-290-5p mimics. Transfected with miR-290-5p mimics, the luciferase enzyme activity had no significant change in the reporter vector containing MUT 3′-UTR of CCL-2 (Figure 5B). We also found that the mRNA (Figure 5C) and protein (Figure 5D) expression of CCL-2 were markedly inhibited in MPC5 transfected with miR-290-5p mimics. As expected, the luciferase activity (Figure 5E), mRNA (Figure 5F), and protein (Figure 5G) levels of CCL-2 were dramatically increased after transfection with miR-290-5p inhibitors.

### Propofol improved LPS-induced MPC5 dysfunction by regulating miR-290-5p/CCL-2 signaling pathway

**Figure 6.**
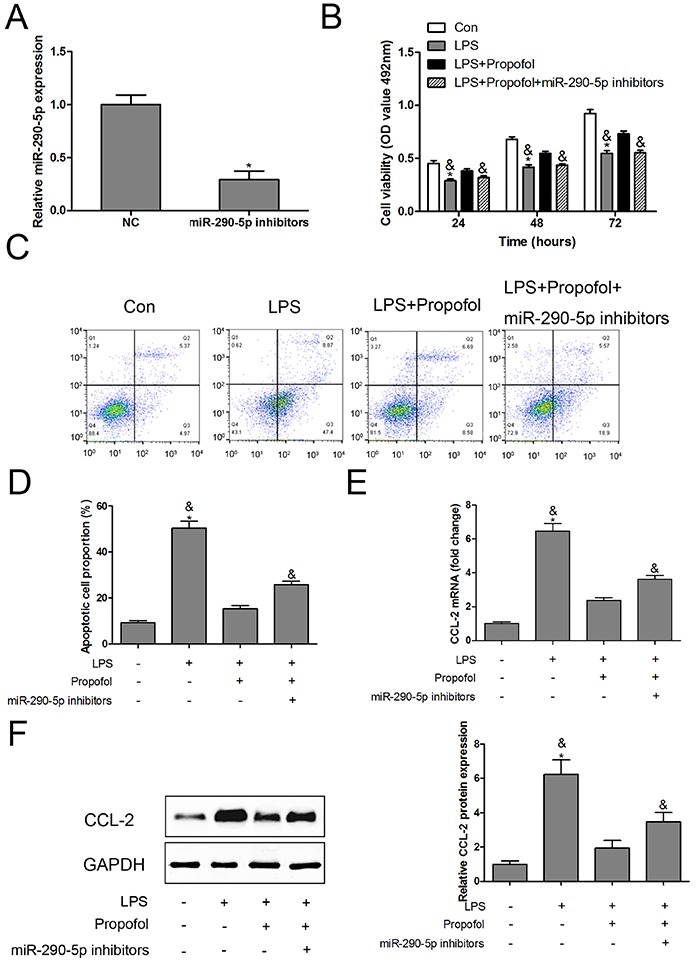
Inhibition of miR-290-5p neutralized the protective effect of propofol in lipopolysaccharide (LPS)-treated mouse podocytes (MPC5). The levels of miR-290-5p were measured by RT-qPCR after being transfected with miR-290-5p inhibitors in MPC5 (*A*). In LPS-treated MPC5 combined with propofol or propofol+miR-290-5p inhibitors, cell viability was measured using the MTS assay (*B*); apoptosis was analyzed using flow cytometry (*C* and *D*); the mRNA (*E*) and protein (*F*) expression of CCL-2 were detected by RT-qPCR and western blotting, respectively. Data are reported as the means±SD (n=3 in each group). *P<0.05 compared with control group; ^&^P<0.05 compared with LPS + propofol group.

To investigate the role of miR-290-5p in LPS and propofol-treated MPC5 podocytes, we found that transfection of miR-290-5p inhibitors significantly down-regulated the expression of miR-290-5p in MPC5 podocytes (Figure 6A). Potential cytotoxicity of LPS and propofol was analyzed using an MTS assay. The results indicated that propofol protected against LPS-induced MPC5 death, however, knockdown of miR-290-5p abrogated the protective effect of propofol on cell viability (Figure 6B). Intriguingly, similar results were obtained by flow cytometry analysis (Figure 6C and D). We also found that knockdown of miR-290-5p abrogated the inhibitory effect of propofol on the mRNA (Figure 6E) and protein (Figure 6F) expression of CCL-2.

## Discussion

It is well known that the inflammatory response plays a central role in the complications that develop in experimental sepsis (27). In septic mice with AKI, CLP leads to formation of oxidative stress, which appears to intensify inflammatory response (13). Suzuki et al. (28) showed that oxidative stress results in an increase in the expression of CCL-2, which accelerates macrophage recruitment, an inflammatory process. Similar mechanisms were seen in our study; we found that CLP induced the expression of CCL-2 and the subsequent increase of the production of proinflammatory cytokines, which might be associated with renal injury in septic mice. Importantly, the post-transcriptional regulatory mechanism demonstrated that miR-290-5p could reversely regulate the expression of CCL-2. The *in vivo* model showed that propofol increased miR-290-5p levels as well as decreased the expression of CCL-2 in CLP-operated mice. The *in vitro* cell model confirmed that propofol protected LPS-induced MPC5 death by inhibiting CCL-2 levels. However, miR-290-5p loss-of-function abrogated the protective effect of propofol on LPS-induced MPC5 apoptosis. All of these findings suggest that propofol can serve as an effective therapeutic medication to suppress sepsis-induced renal injury *in vivo* and *in vitro* by activating miR-290-5p and the subsequent inhibiting CCL-2 and its downstream pathways, such as the inflammatory response.

Propofol has been shown to be capable of anti-inflammatory and anti-apoptotic effects, which may be attributed to its structural similarity to anti-inflammatory medications (10). Consistent with previous studies (11,13), our results indicated that propofol treatment was shown to inhibit inflammatory reaction *in vivo* and attenuate apoptosis *in vitro* by targeting miR-290-5p/CCL-2 signaling pathway. Notably, CLP-treated mice or LPS-treated podocytes have increased expression of CCL-2, suggesting that CCL-2 may be central in the pathological process of renal injury, as reported previously (13,29). We proposed the mechanism for the protective role of propofol, which protected against CLP or LPS-induced renal injury by inactivation of CCL-2 and its downstream inflammatory cytokines.

Further study on molecular mechanisms have examined the effects of propofol on miRs expression. In the present study, propofol significantly increased the expression of miR-290-5p in the kidney from septic mice. miR-290-5p is a member of the miR-290-295 cluster, which are the most abundant miRs and mediate a latent pro-survival function in mouse embryonic stem cells (mESCs) (30,31). miR-290-295 cluster deficiency results in partially penetrant embryonic lethality and germ cell defects in mice (32). In addition, miR-290-295 cluster has been found to accelerate cell proliferation by promoting the G1 to S phase transition (33), suggesting a role for this miR cluster in serving a protective function in preventing mESCs apoptosis. The anti-apoptotic functional consequence of miR-290-5p in LPS-treated podocytes was confirmed by our *in vitro* experiments, which provided evidence that miR-290-5p may decrease the renal injury by suppressing podocytes apoptosis. On the other hand, direct damage to renal tissues or cells by CLP or LPS might be alleviated through miR-290-5p-targeted CCL-2 and inflammatory cytokines. To our knowledge, this is the first study to report the anti-inflammatory activity of miR-290-5p in the animal and cell model of sepsis. Preliminary study has shown that improvement of survival rates and renal damage and inhibition of inflammation by propofol treatment in septic mice may be associated with up-regulation of miR-290-5p. These results suggest that miR-290-5p may be a potential synergist with propofol to increase survival rates in septic mice. A previous study found that miR-125b inserted into a lentivirus expression vector could prevent cardiac dysfunction and improve survival rates (approximately 30%) in septic mice (34). Therefore, we hypothesized that lentivirus-expressed miR-290-5p may be a potential synergist with propofol. However, these conclusions need to be validated by *in vivo* experiments.

There are some limitations in our study. Although our results indicated that propofol inhibited CLP-induced AKI by enhancing miR-290-5p expression, a direct renoprotection of exogenous miR-290-5p in septic mouse model remains undefined. In addition, the regulating effect of CCL-2 on inflammatory cytokines might exist in the progression of sepsis-associated AKI, but the effect of CCL-2 gain-of-function or loss-of-function on inflammatory response is still not elucidated. Thus, the results obtained here only exhibit the possible therapeutic target of propofol in sepsis-induced AKI.

In conclusion, propofol decreased inflammatory cytokines in CLP-operated mice and inhibit apoptosis in LPS-treated podocytes. In addition, propofol also protected the kidney from sepsis-induced injury, and the underlying mechanism was mediated, at least partially, by regulation of miR-290-5p/CCL-2 signaling pathway. These findings indicated that propofol might be used as a promising therapeutic agent for preventing sepsis-induced AKI in routine clinical practice.
